# Co-culture of Schwann cells and endothelial cells for synergistically regulating dorsal root ganglion behavior on chitosan-based anisotropic topology for peripheral nerve regeneration

**DOI:** 10.1093/burnst/tkac030

**Published:** 2022-09-04

**Authors:** Tiantian Zheng, Linliang Wu, Shaolan Sun, Jiawei Xu, Qi Han, Yifan Liu, Ronghua Wu, Guicai Li

**Affiliations:** Key laboratory of Neuroregeneration of Jiangsu and Ministry of Education, Co-innovation Center of Neuroregeneration, NMPA Key Lab for Research and Evaluation of Tissue Engineering Technology Products, Nantong University. 226001, Nantong, P. R. China; Key laboratory of Neuroregeneration of Jiangsu and Ministry of Education, Co-innovation Center of Neuroregeneration, NMPA Key Lab for Research and Evaluation of Tissue Engineering Technology Products, Nantong University. 226001, Nantong, P. R. China; Key laboratory of Neuroregeneration of Jiangsu and Ministry of Education, Co-innovation Center of Neuroregeneration, NMPA Key Lab for Research and Evaluation of Tissue Engineering Technology Products, Nantong University. 226001, Nantong, P. R. China; Key laboratory of Neuroregeneration of Jiangsu and Ministry of Education, Co-innovation Center of Neuroregeneration, NMPA Key Lab for Research and Evaluation of Tissue Engineering Technology Products, Nantong University. 226001, Nantong, P. R. China; Key laboratory of Neuroregeneration of Jiangsu and Ministry of Education, Co-innovation Center of Neuroregeneration, NMPA Key Lab for Research and Evaluation of Tissue Engineering Technology Products, Nantong University. 226001, Nantong, P. R. China; School of Medicine, Nantong University. 226001, Nantong, P. R. China; Key laboratory of Neuroregeneration of Jiangsu and Ministry of Education, Co-innovation Center of Neuroregeneration, NMPA Key Lab for Research and Evaluation of Tissue Engineering Technology Products, Nantong University. 226001, Nantong, P. R. China; Key laboratory of Neuroregeneration of Jiangsu and Ministry of Education, Co-innovation Center of Neuroregeneration, NMPA Key Lab for Research and Evaluation of Tissue Engineering Technology Products, Nantong University. 226001, Nantong, P. R. China; School of Medicine, Nantong University. 226001, Nantong, P. R. China; Guangxi Key Laboratory of Regenerative Medicine, Guangxi Medical University, 530021, Nanning, P.R.China; National Engineering Laboratory for Modern Silk, Soochow University, Suzhou 215123, China

**Keywords:** Schwann cells, Endothelial cells, Dorsal root ganglion behavior, Co-culture, Anisotropic topology, Regulation mechanism, Nerve, Regeneration

## Abstract

**Background:**

Anisotropic topologies are known to regulate cell-oriented growth and induce cell differentiation, which is conducive to accelerating nerve regeneration, while co-culture of endothelial cells (ECs) and Schwann cells (SCs) can significantly promote the axon growth of dorsal root ganglion (DRG). However, the synergistic regulation of EC and SC co-culture of DRG behavior on anisotropic topologies is still rarely reported. The study aims to investigate the effect of anisotropic topology co-cultured with Schwann cells and endothelial cells on dorsal root ganglion behavior for promoting peripheral nerve regeneration.

**Methods:**

Chitosan/artemisia sphaerocephala (CS/AS) scaffolds with anisotropic topology were first prepared using micro-molding technology, and then the surface was modified with dopamine to facilitate cell adhesion and growth. The physical and chemical properties of the scaffolds were characterized through morphology, wettability, surface roughness and component variation. SCs and ECs were co-cultured with DRG cells on anisotropic topology scaffolds to evaluate the axon growth behavior.

**Results:**

Dopamine-modified topological CS/AS scaffolds had good hydrophilicity and provided an appropriate environment for cell growth. Cellular immunofluorescence showed that in contrast to DRG growth alone, co-culture of SCs and ECs could not only promote the growth of DRG axons, but also offered a stronger guidance for orientation growth of neurons, which could effectively prevent axons from tangling and knotting, and thus may significantly inhibit neurofibroma formation. Moreover, the co-culture of SCs and ECs could promote the release of nerve growth factor and vascular endothelial growth factor, and up-regulate genes relevant to cell proliferation, myelination and skeletal development via the PI3K-Akt, MAPK and cytokine and receptor chemokine pathways.

**Conclusions:**

The co-culture of SCs and ECs significantly improved the growth behavior of DRG on anisotropic topological scaffolds, which may provide an important basis for the development of nerve grafts in peripheral nerve regeneration.

Highlights

- Anisotropic CS/AS topology was prepared using micro-molding technology.
- Co-culture of SCs and ECs promoted DRG axon growth and neuron orientation.
- Co-culture of SCs and ECs showed the highest release of nerve growth factor.
- The study provided an important basis for the development of nerve grafts.

## Background

Peripheral nerve injury is a serious disease in clinical medicine, which has diverse pathogenic factors, including trauma, surgical accidents, sports injuries, etc. [1,2]. Although most peripheral nerve injuries do not endanger human life, partial nerve defects hinder functions of normal surrounding tissue and cause action inconvenience and paresthesia to patients, which adversely affects people’s daily life [3,4]. Therefore, the repair of peripheral nerve injury is urgent and plays a vital role in a patient’s life [5]. At present, the common treatment methods for nerve injury mainly include surgical repair, autologous transplantation, tissue engineering technology and gene therapy [6,7]. Among these, tissue engineered implants have shown broad application prospects in the field of peripheral nerve repair due to their good biocompatibility and easy acquisition and processing [8,9]. However, it is still difficult to achieve similar effects to those of autologous implants [10]. Therefore, it is necessary to conduct further explorations on peripheral nerve implants with optimal performance for nerve repair and regeneration [11,12].

It has been reported previously that the surface properties, e.g. the physical topography, of the implants could significantly affect cell growth and tissue regeneration in addition to the bulk properties of the implants per se, and show a great effect on the neural recovery process [13,14]. Moreover, numerous studies have found that the main function of surface topological structure is to effectively guide the orientation growth of the neo-regenerated axons, resulting in accelerated regeneration progress for injured nerves [15,16]. However, the single topology structure of the scaffolds usually has limited ability to promote nerve regeneration due to the lack of sustainable bioactivity, since most of the biomaterial scaffolds are non-vital and thus hinder the long-term survival of nerve cells and weaken the repair effect on the injured nerve.

Endothelial cells (ECs) are a type of phagocytic cell that is widely distributed in the capillary network and has the ability to swallow bacteria, foreign matter and necrotic tissues [17], while the capillary network itself bears the task of transporting nutrients for nerve tissue and participates in the growth of nerve fiber bundles to ensure their normal function *in vivo* [18]. Moreover, ECs were found to have the function of promoting nerve growth and repair [19]. Dorsal root ganglion (DRG) is composed of axons of sensory neurons in spinal ganglia, is responsible for receiving all nerve impulses from body receptors and plays an important role in the modulation of peripheral nerve conduction function. The growth of DRG co-cultured with ECs appeared to be much faster than that of a simple DRG culture in the same cell growth microenvironment, which may have a close relationship with growth factors secreted by ECs or the cell contact made between ECs and DRG [20,21]. Moreover, ECs could enhance the proliferation of DRG and increase the expression of proliferation-related proteins by promoting the secretion of vascular endothelial growth factor (VEGF) and nerve growth factor (NGF) [22,23]. In addition, endothelial progenitor cells were also reported to improve the growth of mouse DRG under co-culture conditions [24].

In the nervous system, glial cells including microglia, astrocytes and Schwann cells (SCs) etc., could support and guide neuronal migration, regeneration and immune response via differentiation and proliferation [25]. SCs are the main glial cells in the peripheral nervous system, which are wrapped in the outer layer of axons and play a key role in the regeneration and repair process of peripheral nerve injury [26,27]. SCs not only promote the growth of neuronal synapses, but also participate in the formation of myelin sheath as functional cells after nerve injury. In addition, SCs are also involved in the formation of membrane structure without myelinated nerve fiber bundles, indicating that SCs are of great significance to the growth of neurons [28,29]. In addition, SCs also secrete a variety of growth factors to promote the growth of DRG, such as NGF, glial cell-derived growth factor, Brain-derived neurotrophic factor (BDNF ) etc. [30,31]. Endo and Richardson *et al*. co-cultured SCs and DRG under different culture conditions and quantitatively measured the length of neurite growth of DRG [32,33]. They found that SCs not only promoted axon growth by promoting NGF secretion, but also directly contacted the protrusions to stimulate DRG growth [34]. Hynds *et al*. further confirmed that the conditioned medium of SCs could also stimulate the secretion of NGF for promoting the growth of DRG neurons. In addition, co-culture of SCs and microglia was also found to obviously synergistically accelerate nerve growth [35,36].

Based on the above, the topological structure provides a physical microenvironment conducive to cell adhesion, proliferation and orientation migration [37],while ECs and SCs have a positive effect on the growth of the DRG and elongation of the axon. Therefore, the synergistic effect of the topological structure and the cell co-culture system may have an additive impact on nerve repair. However, until now, there has been no relevant study on the synergistic effect of ECs and SCs combining with anisotropic topology structure for regulating DRG behavior in the repair process of peripheral nerve injury. In this study, chitosan/artemisia sphaerocephala (CS/AS) anisotropic topological scaffolds were first prepared by a micro-molding method, and the surface of the scaffolds was then modified with dopamine (DA) to enhance cell adhesion. CS has good biocompatibility and anti-inflammatory properties, while AS possesses good viscosity and mechanical properties. After that, various physical and chemical performance characterizations were performed, including morphology, wettability, surface roughness and component variation. Then, SCs and ECs were co-cultured separately and together with DRG cells on CS/AS scaffolds with anisotropic structure, respectively, to evaluate the synergistic impact of EC and SC co-culture on DRG behavior for nerve regeneration. Subsequently, the relevant mechanism was investigated by high-throughput transcriptome sequencing and molecular biology assays. The present study is expected to provide a significant reference for microenvironment construction for regulating DRG behavior and may provide an experimental and theoretical basis for the development of nerve grafts in the field of nerve regeneration.

## Methods

### Materials

Rat RSC96 SC line and rat vascular vein EC line were purchased from Bangchen Co., Ltd, Shanghai. Sprague–Dawley (SD, 1 day) suckling mice of Specific Pathogen Free (SPF) were provided by the Experimental Animal Center of Nantong University. Chitosan was obtained from Xingcheng Biological Products Factory (Jiangsu, China). AS powder was purchased from Ningxia Oasis Grass Industry Co., Ltd. Penicillin–streptomycin, DA, 4′,6-diamidino-2-phenylindole (DAPI), cytarabine and poly-D-lysine were all obtained from Sigma-Aldrich in the USA. Collagenase, trypsin, fetal bovine serum (FBS), 1640 medium, neurobasal medium, Dulbecco’s modified Eagle’s medium (DMEM) and high-glucose basal medium were all purchased from Gibco, USA. Phosphate-buffered saline (PBS, pH = 7) was purchased from HyClone. Paraformaldehyde came from the National Pharmaceutical Group Chemical Reagent Co., Ltd, China. Tris (hydroxymethyl) methyl aminomethane was bought from Sinopharm Chemical Reagent Co., Ltd, China. Immunofluorescence blocking solution, primary antibody diluent and secondary antibody diluent were purchased from Beyotime Biotechnology Co., Ltd, Shanghai. Rabbit monoclonal [4B3] to S100, goat anti-rabbit IgG H&L (Cy3 ®) pre-adsorbed, mouse monoclonal [2G10] to beta III tubulin-neuronal marker and goat anti-mouse IgG H&L (Alexa Fluor 488) were provided by Abcam.

### Preparation of anisotropic topology CS/AS scaffolds

**Figure 1.**
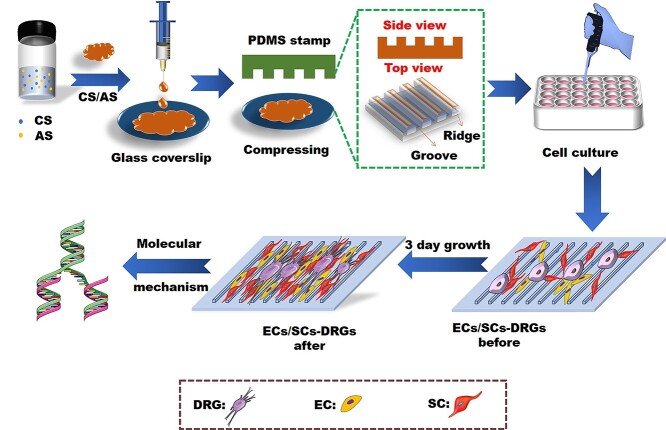
Schematic diagram of the preparation of patterned CS/AS scaffolds for cell co-culture. *CS* chitosan, *AS* artemisia sphaerocephala, *PDMS* polydimethylsiloxane, *DRG* dorsal root ganglion, *EC* endothelial cell, *SC* Schwann cell

The preparation process of anisotropic topology CS/AS scaffolds used in this experiment is shown in Figure 1. CS (2.5 g chitosan powder + 98 ml of pure water + 2 ml of acetic acid) and AS were magnetically stirred at a ratio of 1:10 (1 ml, 10 mg) for 12 h, mixed well and stored in a refrigerator at 4°C. A syringe was used to drop 50 μl of CS + AS mixed solution on the glass coverslips and a polydimethylsiloxane stamp with a groove width of 30 μm was imprinted on the droplet. After 2 days of natural air drying, the stamp was peeled off to obtain a complete patterned CS/AS scaffold (Topo-G group). Then the slide was immersed in 4% sodium hydroxide solution for 30 min for deacidification. After the slide was washed with deionized water to neutral, the samples were treated with DA (2 mg/ml, pH = 8.5) for 24 h, and the incomplete adsorption solution on the slide was washed with deionized water. The sample obtained was named D-Topo-G group, and the pure slide (G group) was treated with DA as a control group (D-G group).

### Morphological observation

First, the four prepared samples were placed on the workbench and dried at room temperature. Then an optical microscope (Leica, Germany) and scanning electron microscope (JEOL, Japan) were used to observe the macroscopic and microscopic morphology and structure of the samples, respectively. In the optical microscopy (OM) test, a pure glass slide was used as the control group, and different magnifications were used to observe the morphology of the samples. In the scanning electron microscopy (SEM) test, the surfaces of the four groups of samples were first sprayed with a gold layer of 50 nm thickness, and then the four groups of samples were fixed on the aluminum plate. The samples were placed in a measurement chamber and observed under the vacuum conditions of 1.4 × 10^−4^ bar. Finally, three regions were selected for each sample and different magnifications of each region were used to observe the sample.

### Atomic force microscopy

An atomic force microscope (FM-Nanoview6800, Flyman Precision Instrument Co., Ltd, China) was used to detect the surface morphology and roughness of the sample. The surface of the sample was scanned by tap mode, the sample to be tested was fixed on the sample platform with double-sided tape, and then the sample was attached to the scanner. The scanning range was set to 6 × 6 μm and the amplitude of the vibration source was set to 0.1 V. The motor was manually controlled to approach the probe. When the distance between the probe and the sample was 1000 nm, five random areas on the sample surface were selected for detection. Finally, the roughness of the sample surface was statistically analyzed.

### Wettability test

The wettability of the sample was tested with a contact angle measurement instrument (JY-PHA, Chengde Jinhe Instrument Manufacturing Co., Ltd China). The larger the contact angle, the more hydrophobic the surface, while the smaller the contact angle, the more hydrophilic the surface. In this experiment, the contact angles of the four groups of samples were measured, including D-Topo-G, Topo-G, D-G and G groups. The steps were as follows. First the sample with diameter of 14 mm was placed on the measurement platform and deionized water (100 μl) was dropped on the surface of the sample and kept for 3 s to achieve balance, and then the measurement was performed using the embedded software. Each sample was made up of a set of five parallel samples, and three independent experiments were performed on each parallel sample. Finally, the average contact angle was calculated by the software.

### Determination by Fourier transformed infrared spectroscopy

The chemical composition of the samples was characterized by a Fourier transform infrared spectrometer (Nicolet iS50, Thermo Fisher) in the wavenumber range 4000–400 cm^−1^. Before the measurement, all the samples (diameter, 14 mm) including D-Topo-G, Topo-G, D-G and G groups were completely dried. Then, the samples were fixed in the measurement chamber and detected using reflective mode. The infrared spectrum was collected by the embedded measurement software.

### Culture of RSC96 SC and EC lines

The RSC96 SC and EC lines were taken out of the −80°C freezer, quickly placed in a 37°C water bath, shaken for 1 min to thaw and then the cells were centrifuged at 1200 rpm for 5 min. After that the cell supernatant was trashed. Then the RSC96 cell pellets were added with 1 ml of culture medium (containing 10% FBS, 4 mM L-glutamine, 100 μg/ml PS and DMEM) and they were then transferred into a Petri dish for incubation. 1640 medium containing 10% FBS, 4 mM L-glutamine and 100 μg/ml PS was added to the EC pellet, which was then transferred to a Petri dish for incubation. Both cells were incubated at 37°C in a 5% carbon dioxide incubator until a bottom coverage of 90% was reached before use.

### Co-culture of SCs, ECs and DRG

All the samples with diameter of 14 mm were first placed into a 24-well culture plate and then pre-sterilized using 75% ethanol for 30 min and washed with PBS three times before cell culture. After that, the 1-day-old SD rat suckling mice were disinfected with 75% ethanol, and then the head was cut, the skin was opened from the back with ophthalmic scissors, the medullary cavity was opened along the spine and the spinal cord was exposed using a microscope. DRG was removed from both sides of the spinal cord by micro tweezers, and DRG was placed in DMEM after nerve roots and capsule were removed. The supernatant was gently aspirated and the DRG explants were seeded onto the samples pre-coated with poly-D-lysine in a 24-well plate, then 2 ml of DRG medium (neurobasal medium + 1:50 B27(Serum-free supplement for nerve cell culture) + 1:1000 NGF + 1: 100 L-glutamine + 1: 100 PS, NGF: 50 ng/ml) was added and cultured in a 37°C, 5% carbon dioxide incubator. The DRG regular medium was replaced every other day. On the third day, 200 μl of SC or EC suspension with a density of 3000 cells/well was seeded onto the samples that had been pre-seeded with DRG explants to obtain SC-DRG and EC-DRG co-culture systems, respectively. For the SC-EC-DRG co-culture system, SCs and ECs were first mixed in equal amounts and then the cell suspension with a cell density of 3000 cells/well was added on to the samples. After co-culture in an incubator at 37°C for 3 days, the cells were fixed with 4% formaldehyde for further analysis. Three parallel samples were set up for each group, and the experiment was repeated three times.

### Cellular immunofluorescence staining

At each time point, freshly prepared 4% paraformaldehyde solution was added into the culture plate for 45 min, and then the sample was washed three times with PBS for 10 min each time. Cells were permeabilized for 10 min with 500 μl of 0.5% Triton X-100 (prepared in PBS, PBST). Immunofluorescence blocking solution was then added to the wells and blocked for 1 h at room temperature, and then the sample was washed three times with PBS for 5 min each time. After that, 200 μl of antibody diluted with primary antibody diluent at a ratio of 1:200 was added (Tuj-1-labeled neuronal cells, S100-labeled SCs and CD31-labeled ECs) and incubated overnight at 4°C. Then, 500 μl of PBST was added to each well and washed three times for 5 min each time. Thereafter, 200 μl of biotin-labeled goat anti-mouse secondary antibodies (SCs) and goat anti-rabbit secondary antibodies (ECs) diluted with secondary antibody diluent at a ratio of 1:200 was added to the culture plate and incubated in the dark at 37°C for 2 h. The nucleus was labeled and stained with DAPI (concentration 2 μg/ml) in the dark at room temperature for 15 min. After washing with 500 μl of PBST per well, 200 μl of fluorescent fixative were added to each well. To prepare the fluorescence fixative, 0.689 g of anhydrous sodium carbonate (Na_2_CO_3_) and 1.554 g of sodium bicarbonate (NaHCO_3_) were added to double distilled water to 50 ml, the pH was adjusted to 9.5 and then 50 ml of non-fluorescent Analytic Reagent (AR) grade glycerin was added. Finally, the cells were observed and photographed using an optical microscope (Zeiss). The length of DRG axons in the co-culture system was measured by Zeiss microscope under 5X and 10X objective lenses respectively.

### Enzyme-linked immunosorbent assay

An enzyme-linked immunosorbent assay was used to detect secreted growth factor, including NGF and VEGF, in the co-culture system. After 48 h of co-culture, 50 μl of supernatant of conditioned medium was harvested for the enzyme-linked immunosorbent assay, 40 μl of sample diluent was added to the sample well of the enzyme-labeled plate and 10 μl of the sample was used for testing. Then 50 μl of enzyme-labeled reagent was added to each well. After sealing the plate with a sealing film it was further incubated at 37°C for 30 min and washed five times with washing solution. Then, 50 μL of chromogenic fluid A was added to each well followed by 50 μL of chromogenic fluid B, shaken gently to mix and then developed in the dark at 37°C for 15 min. Finally, 50 μl of stop solution was added to each well to stop the reaction. The Benchmark-Plus Reader (Bio-Rad, Hercules, California, USA) was used to measure the absorbance of each well at 450 nm. Five parallel samples were set up for each group.

### RNA sequencing

After co-culture of SCs, ECs and DRG for 3 days, the total RNA of each sample was extracted by Trizol Reagent, RNeasy Mini Kit (Qiagen) or other kits. There were four groups of samples. Three types of cells were cultured on pure glass slides as the control group (G-control or R-E-G group) and the other three groups were experimental groups. SCs or ECs and DRG were co-cultured on topological structure (R-D-T group, E-D-T group, respectively), and three kinds of cells were co-cultured on topological structure (R-E-T group). The total RNA of each sample was passed through an Agilent 2100/2200 biological analyzer (Agilent Technologies, Palo Alto, CA, USA) and NanoDrop (Thermo Fisher Scientific Inc). Total RNA (1 μg) was used for subsequent library preparation. According to the protocol of the manufacturer, the next-generation sequencing library preparation was constructed and sequenced on the RNA-seq platform. Genewiz Biotechnology Co., Ltd (Suzhou, China) conducted RNA library construction and sequencing.

**Figure 2.**
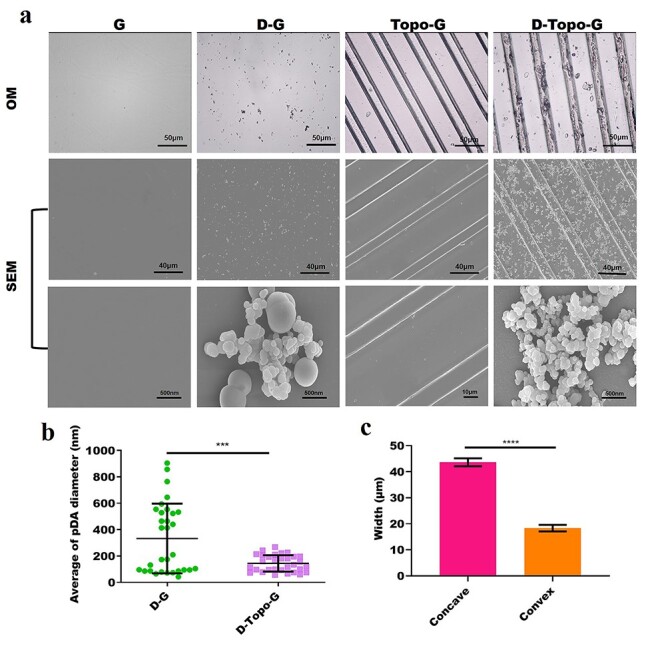
Morphological characteristics of the patterned CS/AS scaffolds. (**a**) Surface topography taken by optical microscope and scanning electron microscope. (**b**) Quantitative analysis of the particle diameter of surface dopamine, *n* = 30. (**c**) Quantitative analysis of concave and convex width of topological structure. Data are shown as means ± SD. Statistical analysis: ^***^p < 0.001, ^****^p < 0.0001. *CS* chitosan, *AS* artemisia sphaerocephala, *SD* standard deviation, *OM* the optical microscopy*, SEM* scanning electron microscopy*, G* glass group, *D-G* the pure slide (glass group) treated with dopamine, *Topo-G* the pure slide treated with topological structure, *D-Topo-G* the pure slide was treated with topological structure with dopamine coating

**Figure 3.**
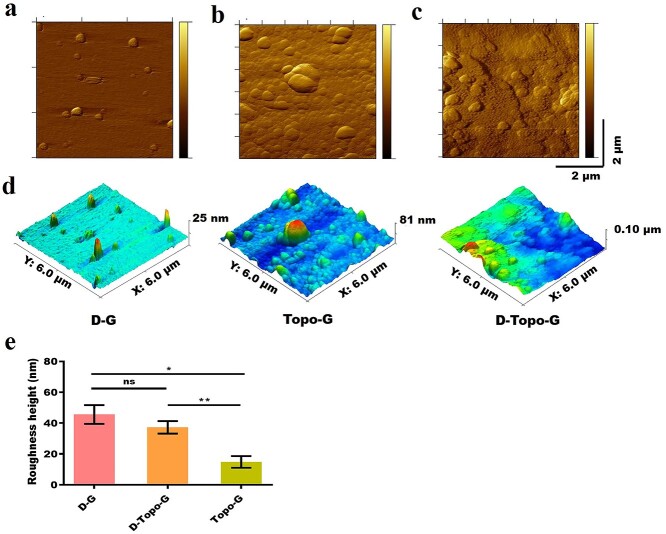
Atomic force microscopy of patterned CS/AS scaffolds. (**a**) Dopamine particles. (**b**) Topological structure. (**c**) Topological structure with dopamine coating. (**d**) 3D structure of samples. (**e**) Quantitative analysis of surface roughness, *n* = 5. Data are shown as means ± SD. One-way analysis of variance was used. Statistical analysis: ^*^p < 0.05, ^**^p < 0.01, *ns* no significance. *CS* chitosan, *AS* artemisia sphaerocephala, *SD* standard deviation, *D-G* the pure slide was treated with dopamine, *Topo-G* the pure slide was treated with topological structure, *D-Topo-G* the pure slide was treated with topological structure with dopamine coating

### Quantitative real-time polymerase chain reaction

SCs, ECs and DRG were co-cultured on the scaffold for 3 days. There were six groups of samples, in addition to R-D-T, E-D-T, R-E-T and R-E-G, the other two groups were R-D-G and E-D-G (SCs or ECs and DRG were co-cultured on pure glass slides, respectively). Trizol (1 ml) was added to the cells for treatment and placed on ice for 5 min. The liquid was pipetted and mixed well, and then 0.2 ml of chloroform was added. The mixture was shaken and mixed for 15 s and then placed on ice for 5 min. The liquid was centrifuged at 12000 rpm for 15 min at 4°C. Isopropanol (500 ml) was added to the supernatant, mixed upside down, and allowed to stand for 10 min. Centrifugation was performed at 12000 rpm at 4°C for 20 min. After discarding the supernatant, 75% ethanol was added to suspend precipitation. Centrifugation was performed at 12000 rpm at 4°C for 15 min. After the supernatant was discarded, the residual was absorbed by a pump to separate the total RNA. PCR primers were synthesized by Shanghai Jierui Bioengineering Co., LTD. (China) (Table S1, see online supplementary material). Subsequently, RNA (500 ng) reverse transcription of single-stranded cDNA was performed using oligonucleotide primers via PrimeScript RT Master Mix. Gene expression was analyzed by FastStart Universal SYBR Green Master in a Step One real-time PCR detection system. mRNA was standardized by using glyceraldehyde-3-phosphate dehydrogenase as a housekeeper gene. The relative gene expression was calculated by comparing 2^−ΔΔCT^ method. Three parallel samples were set up for each group, and the experiment was repeated three times.

### Statistical analysis

The data in this study were analyzed using Origin 9.1 (Origin Lab), GraphPad prism 8 and Image J software. A *t*-test was used for comparison between two groups. Multigroup comparisons of the means were carried out by one-way analysis of variance with *post hoc* contrasts by Tukey test. The results are expressed as the mean value (x̅) ± standard deviation (SD). A probability (*p*) value < 0.05 (*p* < 0.05) was considered as significant difference. In the figures, we use ^*^ to denote *p* values and ns to denote no significance, in which ^*^p < 0.05, ^**^p < 0.01, ^***^p < 0.001 and ^****^p < 0.0001.

## Results

### Morphological analysis of the film

As shown in Figure 2a, the surface morphology of the CS/AS patterned scaffolds was observed by OM and SEM. DA was adsorbed on the surface of the glass slide or topological structure to form a DA coating, which presented as gray–brown spherical suspended particles under OM. Figure 2b shows that DA aggregated on a pure glass slide to form a polymer, but was dispersed in the topological structure. The size of the DA particles on the pure glass slide was significantly larger than the size of the DA particles on the topological structure. After the polydimethylsiloxane stamp was imprinted, the film surface had an obvious ridge/groove topology structure. Figure 2c shows that the width of the concave surface was 46 μm, the width of the convex surface was 18 μm and the width of the concave surface was approximately three times that of the convex surface. This structure was conducive to cell growth along the topological structure.

### Atomic force microscopy

Figure 3 shows the surface morphology and roughness of the film observed by an atomic force microscope. Figure 3a and c show that DA particles were randomly deposited on the surface of the glass slide to form a coating. The CS/AS hybrid film on the surface of the glass slide is shown in Figure 3b, and 3D images of the three sets of samples are shown in Figure 3d. Quantitative analysis was performed on the surface roughness of the three groups of samples. As shown in Figure 3e, the roughness of the D-G and D-Topo-G groups was 45.62 and 37.3 nm, respectively, and there was no significant difference in the roughness of the two groups. While the roughness of Topo-G group was 14.886 nm, the roughness of D-G and D-Topo-G groups was obviously greater than that of Topo-G group, indicating that DA adhered to the surface of the material dominated the surface properties of the material. 

**Figure 4.**
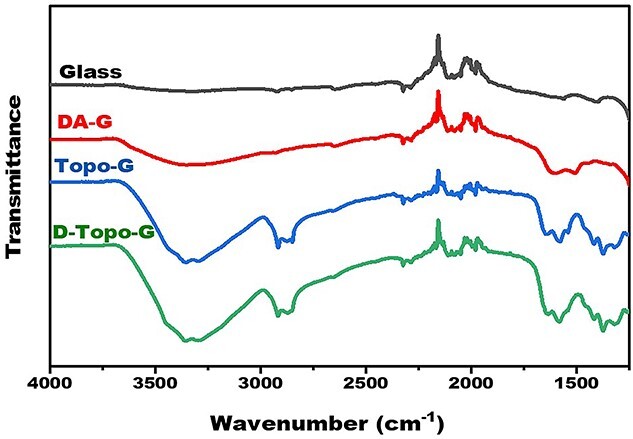
Infrared spectrum of the patterned CS/AS scaffolds. *CS* chitosan, *AS* artemisia sphaerocephala, *G* glass group, *D-G* the pure slide was treated with dopamine, *Topo-G* the pure slide was treated with topological structure, *D-Topo-G* the pure slide was treated with topological structure with dopamine coating

### Infrared spectrum analysis

As shown in Figure 4, infrared spectroscopic analysis was performed on four groups of samples. Amino and catechol are the highly chemically active molecular structures of DA. Amino groups are also important functional groups in chitosan. Compared with the pure glass slide group, the absorption peak at 1600 ~ 1450 cm^−1^ after DA treatment was due to vibration of the C=C bond in the aromatic compound DA. In addition, there was an absorption peak at 3308 cm^−1^, which was ascribed to absorption of N–H bond stretching vibration in DA, indicating that DA was bound to the surface. At 3000 cm^−1^, the peaks in the Topo-G and D-Topo-G groups were due to the C–H stretching vibration on the aromatic ring. The absorption peak at 2920–2850 cm^−1^ was caused by the C–H stretching vibration of chitosan alkanes. The results also proved that the samples were successfully modified by DA.

### Contact-angle measurement

**Figure 5.**
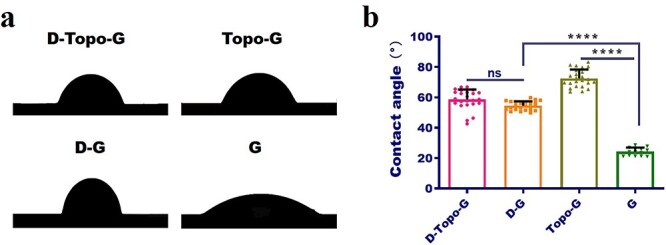
Wettability of the CS/AS scaffolds. (**a**) Physical image of the contact angle. (**b**) Quantitative analysis of the contact angle, *n* = 20. Data are shown as means ± SD. Statistical analysis: ^****^p < 0.0001, *ns* no significant difference. *CS* chitosan, *AS* artemisia sphaerocephala, *SD* standard deviation, *G* glass group, *D-G* the pure slide was treated with dopamine, *Topo-G* the pure slide was treated with topological structure, *D-Topo-G* the pure slide was treated with topological structure with dopamine coating

The wettability variation as a function of DA modification was monitored using contact-angle measurement. Figure 5a shows the photographs of the contact angle of the four groups of samples, which are statistically analyzed in Figure 5b. The results show that the contact angles of the four groups of samples were all <90°, which proved that the samples used in this experiment were all hydrophilic. The average contact angle of the Topo-G group was 72.57°. In contrast, the contact angles of the other three groups were smaller and the hydrophilicity was higher. The minimum contact angle of the pure slide group was 24.3°, indicating the highest hydrophilicity, while the contact angles of D-Topo-G and D-G groups were 58.60 and 54.56°, respectively; there was no significant difference between the two groups. The results indicated that DA modification could alter the hydrophilic ability of the sample.

### Co-culture system promotes DRG neuron axon growth

**Figure 6.**
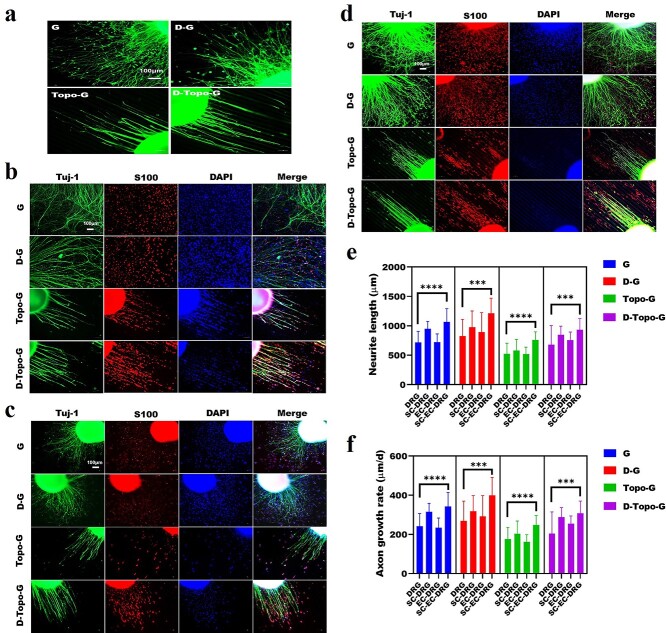
Cell co-culture system promotes the growth of DRG axons. Immunofluorescence staining of (**a**) DRG cultured alone; (**b**) SCs and DRG co-culture; (**c**) ECs and DRG co-culture; (**d**) SCs, ECs and DRG co-culture (scale bar, 100 μm). (**e**) Quantitative analysis of the mean axon outgrowth length of DRG, *n* = 22. (**f**) Quantitative analysis of the mean axon outgrowth rate of DRG, *n* = 22. Data are shown as means ± SD. One-way analysis of variance was used. Statistical analysis: ^***^p < 0.001, ^****^p < 0.0001. *CS* chitosan, *AS* artemisia sphaerocephala, *SD* standard deviation, *G* glass group, *D-G* the pure slide was treated with dopamine, *Topo-G* the pure slide was treated with topological structure, *D-Topo-G* the pure slide was treated with topological structure with dopamine coating, *DRG* dorsal root ganglion*, EC* endothelial cell*, SC* Schwann cell

In order to explore the interaction between SCs, ECs and DRG, we carried out a new co-culture system of different cells. In this system, three co-culture systems were set up: SC-DRG , EC-DRG and SC-EC-DRG. The DRG explants from suckling mice were first cultured on the four groups or samples for 3 days, and then SCs and ECs were co-cultured separately or with DRG. Contact between SCs, ECs and neuron cell bodies could be easily identified by co-labeling with S100 (red), CD31 (red) and Tuj-1 (green), and nuclei were identified by DAPI (blue). Figure 6a shows the morphology of a single DRG; the cell soma has shorter axons and lower axon density. Nevertheless, as shown in Figure 6b–d, when DRG was co-cultured with SCs or ECs on the four samples, the growth of DRG axons was better than that of DRG alone in the different groups. The quantitative statistics of the DRG axon outgrowth length and rate of each group (Figure 6e, f), show that the length and rate of the DRG axon cultured separately in the G and D-G groups was longer than that in the Topo-G and D-Topo-G groups. The longest length of the pure DRG axons cultured on the four sets of samples was only 987.08 μm. When SCs or ECs were co-cultured with DRG, the length of the axons emitted by the cell soma became longer on the four samples. However, compared with the EC-DRG and SC-DRG co-culture systems, the co-culture of SC-EC-DRG had a greater effect on the growth of DRG axons. When SCs were co-cultured with DRG, the longest axon length from cell soma on the four samples was 1483.59 μm. When ECs and DRG were co-cultured, the longest axon length from cell soma on the four samples was 1395.33 μm. Interestingly, when SCs were co-cultured with ECs and DRG, the cell soma emits the longest axon in the same culture time, and the longest axon could reach 1743.32 μm.

**Figure 7.**
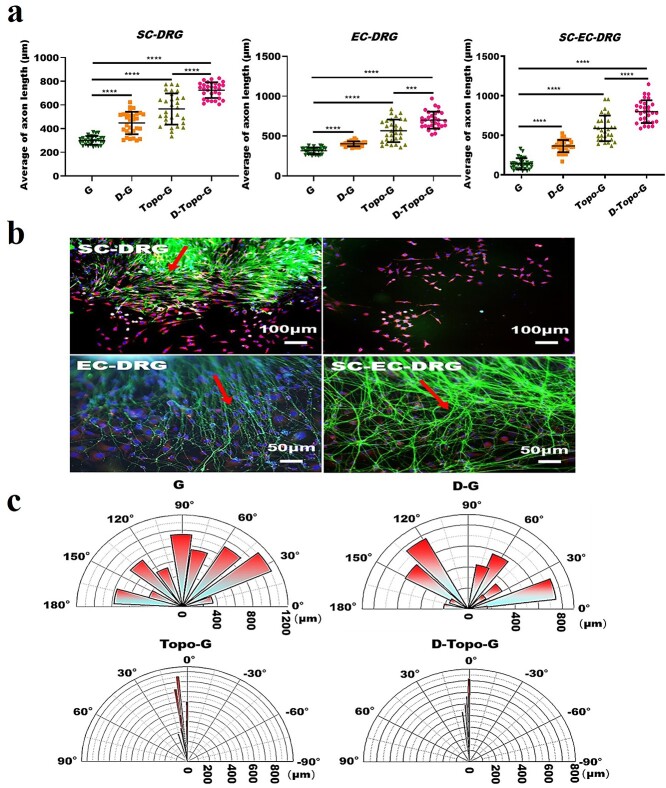
Cell-oriented growth on topological structure. (**a**) Quantitative analysis of unknotted axon outgrowth length in SC-DRG, EC-DRG and SC-EC-DRG co-culture systems, *n* = 30. (**b**) Top panels: the cell growth status under low magnification when SCs and DRG are co-cultured on the glass group (scale bar, 100 μm). Left image: SCs around neurons were observed to grow along the axon poles, while they grew in circles at the end of the axons. However, SCs grew in multipolarity when they were far away from the DRG soma (right image). Below: cell entanglement under magnification when EC-DRG and SC-EC-DRG are co-cultured on the glass group (scale bar, 50 μm). The red arrows indicate the axon outgrowth orientation. (**c**) Quantification of cell orientation angle in the SC-EC-DRG co-culture system on the samples of the four groups. Data are shown as means ± SD. One-way analysis of variance is used. Statistical analysis: ^***^p < 0.001, ^****^p < 0.0001. *SD* standard deviation, *G* glass group, *D-G* the pure slide was treated with dopamine, *Topo-G* the pure slide was treated with topological structure, *D-Topo-G* the pure slide was treated with topological structure with dopamine coating, *DRG* dorsal root ganglion*, EC* endothelial cell, *SC* Schwann cell

**Figure 8.**
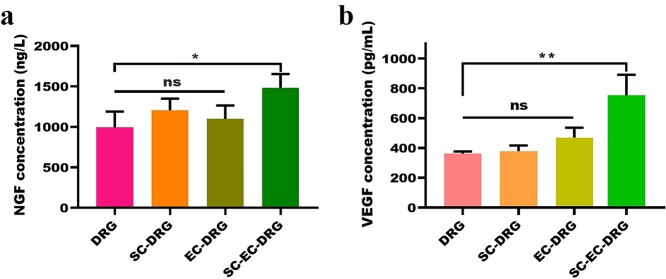
Determination of NGF and VEGF concentration in cell culture medium by enzyme-linked immunosorbent assay, *n* = 3. The method of one-way analysis of variance is used. Data are shown as means ± SD. Statistical analysis: ^*^p < 0.05, ^**^p < 0.01, *ns* no significant difference. *SD* standard deviation, *EC* endothelial cell, *SC* Schwann cell, *NGF* nerve growth factor, *VEGF* vascular endothelial growth factor

**Figure 9.**
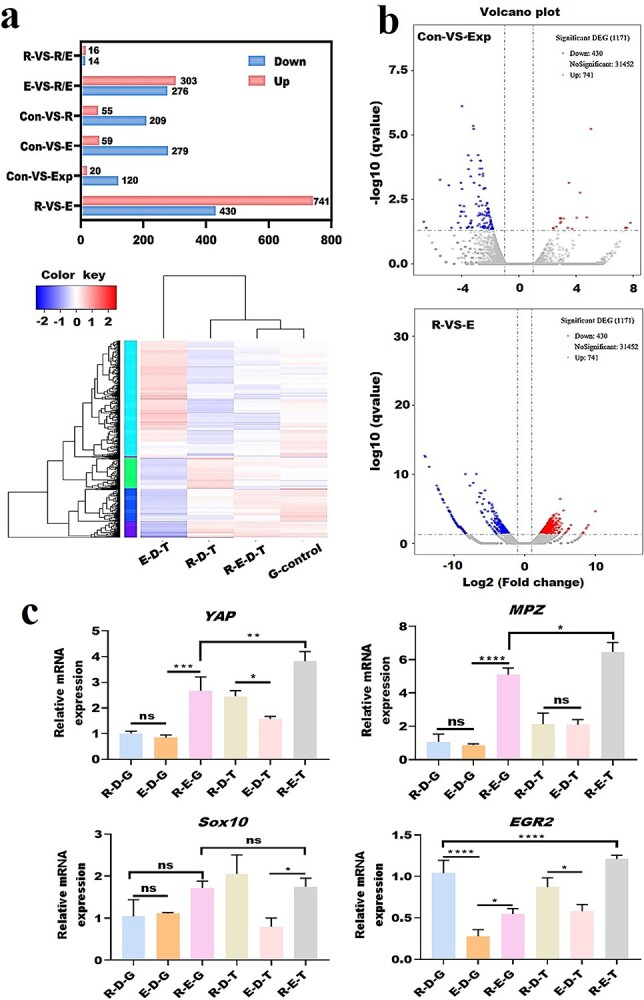
Gene expression of cells co-cultured on patterned CS/AS scaffolds. (**a**) Transcriptome sequence. Upper panel: up-regulated/down-regulated genes in cells. Lower panels: heat map of differentially expressed genes in different groups. (**b**) Volcano map of differentially expressed genes; red dots indicate up-regulation and blue dots indicate down-regulation. (**c**) Gene expression detected by quantitative real-time PCR. One-way analysis of variance is used. Data are shown as means ± SD. Statistical analysis: ^*^p < 0.05, ^**^p < 0.01, ^***^p < 0.001, ^****^p < 0.0001, *ns* no significant difference. *CS* chitosan, *AS* artemisia sphaerocephala, *SD* standard deviation, *R* RSC96 rat Schwann cell, *E* endothelial cell, *Con* control group, *Exp* experiment group, *D* dorsal root gGanglion, *G* glass, *T* topological structure, *YAP* yes-associated protein, *MPZ* myelin protein zero, *Sox10* SRY-box transcription factor 10, *EGR2* early growth response 2

**Figure 10.**
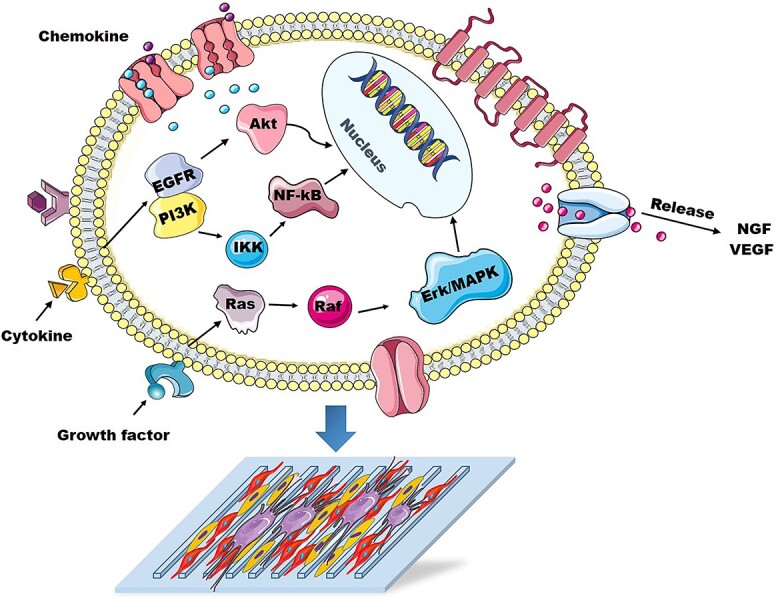
Summary of the possible influence mechanism of anisotropic topological structure and cell co-culture on RSC96 cells. *NGF* nerve growth factor, *VEGF* vascular endothelial growth factor, *Akt* protein kinase B, *NF-kB* nuclear factor kappa-B, *EGFR* epidermal growth factor receptor, *PI3K* the phosphoinositide 3-kinase, *IKK* inhibitor of nuclear factor kappa-B kinase, *MAPK* mitogen-activated protein kinase, *Erk* extracellular signal-regulated kinase

### Oriented cell growth behavior

Figure 7a shows the quantitative analysis of the length of the axons. It can be seen that no axon twist was observed when the cells were co-cultured on the four samples. When SCs and DRG were co-cultured on the four groups of samples, the average length of the axons that were not twisted on the glass slide without any modification was 301.22 μm, whereas it was 447.76 and 565.89 μm on the samples modified by DA and topology, respectively. However, an axon length of 724.78 μm was achieved on the topological structure modified by DA when SC-DRG were co-cultured. When ECs and DRG were co-cultured on the samples, the average length of the axons that were not twisted was 316.53 μm on the glass slide without any modification. The average lengths of axons on the glass coverslip modified by DA and topology were 403.01 and 566.48 μm, which however was 700.79 μm on the topological structure modified by DA for EC-DRG co-culture. Furthermore, when SCs, ECs and DRG were co-cultured, the average length of the axons that were not twisted on the glass slide without any modification was 106.27 μm, and 360.84 and 587.68 μm when modified by DA and topology; however the average length was 799.28 μm on the topological structure modified by DA with SC-EC-DRG co-culture. The results show that the axon growth on the D-Topo-G group was significantly better than that of the other three groups. As shown in Figure 7b, after SCs and DRG were co-cultured on the glass group for 3 days, SCs around neurons were observed to grow along the axon poles, while they grew in circles at the end of the axons. However, SCs grew in multipolarity when they were far away from the cell soma. Interestingly, when cells were co-cultured on a glass slide without any modification, the axons emitted by the DRG explants would become knotted and entangled after a period of straight growth. The knotting and entanglement of DRG axons is because the neurofilaments from the neuron cell body are entangled with each other, which shortens the length of the neurofilaments and affects the growth of DRG axons. After modification with DA, the axon winding phenomenon was reduced. On the glass slide with topological structure, neuron axons grew oriented along the topological structure (Figure 6). As shown in Figure 7c, statistical analysis of the orientation angle of the cells showed that the cells in the G and D-G groups had no obvious orientation when the three types of cells were co-cultured, while the cells in the Topo-G and D-Topo-G groups showed obvious orientation growth behavior, and most of the cells grew along the topological structure; the D-Topo-G group had better cell orientation than the Topo-G group.

### Growth factor release

In order to explore the possible reason for the co-culture behavior of SCs, ECs and DRG, the concentrations of growth-promoting factors NGF and VEGF in the culture medium were determined. As shown in Figure 8, the concentrations of NGF and VEGF in pure DRG culture were only 991 ng/l and 361.67 pg/ml. However, when SCs or ECs were co-cultured with DRG, the concentration of NGF in the culture medium increased to 1205.7 and 1098.55 ng/l, respectively, and the concentration of VEGF increased to 378.33 and 470 pg/ml, respectively. Notably, the concentrations of NGF and VEGF reached 1532 ng/l and 753.33 pg/ml in the SCs-ECs-DRG co-culture system, which were the highest among all the samples.

### Related gene expression

As shown in Figure 9, and Figures S1 and S2 (see online supplementary material), transcriptome sequencing and quantitative real-time polymerase chain reaction (qRT-PCR) were used to detect differences in the expression of cell-related genes under the synergistic effect of cell co-culture and topological structure. Figure 9a shows that compared with the G-control group, the other three groups displayed down-regulated expression of 20 genes and up-regulated expression of 120 genes. The R-D-T group had 55 down-regulated genes and 209 up-regulated genes, while the E-D-T group had 59 down-regulated genes and 259 up-regulated genes compared with the G-control group. The volcano graph in Figure 9b shows that after Edger analysis of RNA sequencing, 741 genes were down-regulated and 430 genes up-regulated between the R-D-T and E-D-T groups. By analyzing the biological functions of all up-regulated and down-regulated genes, we selected several genes related to axon growth, cell proliferation, migration and myelination, such as contact protein, SRY-box transcription factor 10 (Sox10), early growth response 2 (EGR2), tumor necrosis factor, TAZ, yes-associated protein (YAP), myelin protein zero (MPZ) and Met, etc. for qRT-PCR analysis. The results in Figure 9c and Figure S2 show that the gene expression of YAP, EGR2, MPZ and Met genes was higher than that of other groups in the co-culture of the three kinds of cells on the topological structure. Compared with co-culture of the three kinds of cells on pure glass slides, co-culture of the three cells on the topological structure had higher expression of YAP, EGR2, Met, Cntn2, tumor necrosis factor-α and MPZ genes. Sox10 gene expression was highest in the R-D-T group. TAZ gene expression in the R-D-G group was the highest, but there was no significant difference in TAZ expression between the R-E-G, R-D-T, E-D-T and R-E-T groups. The results of gene expression showed that the synergy of cell co-culture and topological structure could lead to the obvious expression of genes related to myelination, cell proliferation and migration.

## Discussion

Peripheral nerve injury is currently a common clinical disease. With the continuous development of tissue engineering and regenerative medicine, the repair of peripheral nerve injury has also been diversified from autologous nerve transplantation to tissue engineered implants [38]. However, the performance of tissue engineered implants needs to be further improved. It is well known that microtopology can regulate and promote nerve regeneration, but nerve fibers that have grown to a certain length would not continue to grow if only guided by the topological structure, which thus hindered the further application of topological biomaterials [39]. Related studies have found that cell co-culture has a positive regulatory effect on the growth behavior of DRG, which may be beneficial for promoting peripheral nerve regeneration. However, at present, most of the research mainly focuses on co-culture systems using two different kinds of cells and revealing their cross-interaction, while few refer to the co-culture of three or more cells. Both ECs and SCs showed a positive influence on DRG growth, but no study on the effect of co-culture of ECs and SCs on DRG growth on an anisotropic structure has been reported, and the specific impact of this co-culture system on nerve repair is currently still unclear. In this study, we first constructed DA-modified CS/AS scaffolds with anisotropic topological patterns using micro-molding technology, and then co-cultured SCs, ECs and DRG on topological scaffolds to evaluate the synergistic effect of ECs and SCs on DRG behavior. We then further investigated the possible underlying mechanism. The results confirmed that the topology structure could direct the orientation growth of DRG axons and enhance their elongation and promote the migration and proliferation of SCs. Moreover, the co-culture of SCs and ECs could improve the growth of DRG axons on topological scaffolds by promoting the release of NGF and VEGF, which may be promising for promoting the regeneration and repair of injured nerves. The co-culture of SCs and ECs could promote cell proliferation, migration and the expression of myelin formation and other related genes. In addition, three possible signaling pathways for the synergistic effect of cell co-culture and scaffold to affect peripheral nerve regeneration were mainly proposed, including the phosphoinositide 3-kinase–protein kinase B (PI3K-Akt) pathway, the mitogen-activated protein kinase (MAPK) pathway and the cytokine and receptor chemokine pathway (Figure 10).

The micropatterned structure affects the morphology of cells, stress fibers and their adhesion ability. The concave surface of the micropattern can promote cell migration, while the convex surface induces cell differentiation [40]. After the material was patterned by micro-molding technology, the hydrophilic properties, cell adhesion and the ability to induce nerve growth could be further improved. In this experiment, DA was first used to modify the topological scaffolds. As DA is a mussel adhesion protein that can adsorb on the surface of any material, it is widely used for surface modification of biological materials [41]. Our previous study proved that DA -modified patterned hydrogels could better promote the directional growth of SCs and had good biocompatibility [42]. In this study, it was found that the CS/AS scaffolds modified by DA had a more obvious promotion effect on the growth and adhesion of DRG compared with the film without DA modification. Thus, DA modification may further improve the potential application of the scaffolds [43].

Previous studies have found that topological structure could promote nerve growth and repair, but this effect was limited, especially for peripheral nerve defects with long gaps [19]. Therefore, it needs to be combined with other factors to better improve the performance of nerve implants. The surrounding vascular microenvironment and neurotrophic factors contribute to the growth and development of the nervous system. The co-culture of neuronal cells and ECs showed increased neuronal division [44]. The SC topographical features could guide the growth of DRG axons [45]. In this experiment, ECs, SCs and DRG were co-cultured on topological biomaterial scaffolds. The results showed that the SCs adjacent to the DRG explant showed a radioactive distribution that grew along the DRG axon direction. Nevertheless, our results showed that the SCs at the end of the axon of the DRG were in a circular distribution around the whole explant, and the SCs far away from the neuron even appeared to exhibit multi-polarization, which was rarely reported in the previous studies. Studies on cell polarity have reported that segmentation defect (PAR) proteins played a vital role in regulating cell polarity. The radial distribution of SCs around DRG may be caused by growth factors secreted by neurons that have a certain affinity for cells. As for the multi-polarization phenomenon of SCs away from DRG axons it may be that the growth factors secreted by neurons cause SCs to distribute in adjacent areas that have sufficient growth factors to promote their growth. On the other hand, for the distal SCs at the end of axons, because of a lack of cell growth factors to stimulate cell proliferation and growth, the genes and protein types may be changed, to e.g. myelin sheath protein, which plays an important role, eventually resulting in the multi-polarization phenomenon of SCs [44]. When SCs, ECs and DRG were co-cultured on the samples of the four groups, the average length of DRG axons was longer than with single SCs or ECs co-cultured with DRG. However, the co-culture of SCs, ECs and DRG on unpatterned samples resulted in axon adhesion and entanglement, which may cause swelling of the patient’s nodules in the actual application process, and further lead to neurofibromas or other undesirable complications. Interestingly, in contrast to co-culture on unpatterned samples, DRG axons grew well after the three types of cells were co-cultured on topological scaffolds, and there were no adhesions and tangles between axons in the later stage, which may indicate the reduced occurrence of neurofibromas in the process of nerve regeneration. However, more work needs to be done to verify the above findings. The results here strongly prove that the synergistic effect of micro-molding technology and EC and SC co-culture are a promising prospect for promoting the oriented growth of neuronal axons and preventing axon entanglement [46].

According to our study, it was also found that the length and rate of DRG axon outgrowth under co-culture of SCs or ECs were much better than in other groups. Previous studies have shown that SCs participated in the formation of axon myelination and secreted NGF to promote nerve growth [36]. ECs also secrete VEGF and NGF to promote the growth of DRG axons. The survival and differentiation of DRG depend mostly on neurotrophic factors, mainly NGF [47]. The results of our study also confirmed that the NGF and VEGF content produced under the conditions of co-culture of SCs and ECs together was higher than that of the other groups. Therefore, the co-culture of SCs and ECs led to an obvious growth of DRG axons. In addition, NGF also has a certain promoting effect on the growth and proliferation of SCs, which may further accelerate the growth and repair of nerves [48].

In addition, after transcriptome sequencing and PCR detection, the possible influence mechanism of the synergistic effect of anisotropic topology and cell co-culture on cell behavior and physiological functions were summarized. In the system of co-culture of SCs, ECs and DRG on the anisotropic CS/AS topology, cells first came into contact with the topology and cell adhesion molecules through different receptors on the cell membrane. After that, the cells received physiological and biological signals, which caused changes in the receptors on the cell membrane. This change was be further transmitted to the interior of the cell through various transmembrane proteins and ion channels. Then, the signal pathway was activated, and the related genes were sequentially up-regulated or down-regulated. Finally, the cells may release growth factors related to cell function, such as NGF and VEGF. Based on the results of transcription sequencing analysis, we proposed three possible signaling pathways: the PI3K-Akt, MAPK and cytokine and receptor chemokine pathways [48,49]. After PI3K bound to epidermal growth factor receptor, it could change the structure of Akt protein to activate it. The activated Akt then activated or inhibited the activity of downstream related proteins, thereby regulating cell proliferation, differentiation and apoptosis. In addition, PI3K could activate the transcription factor inhibitor of nuclear factor kappa-B (IkB) kinase, which could be phosphorylated into IkB-α protein after activation. This meant that IkB-α became detached from nuclear factor kappa-B (NF-kB), and the detached NF-kB was transferred to the nucleus and combined with DNA, resulting in changes in cell function. When GTP replaced GDP and bound to Ras, phosphorylation activated the downstream pathway Raf, which then activated the serine kinase cascade, thus finally activating extracellular signal-regulated kinase (Erk)/MAPK, thereby regulating cell adhesion and preventing cell apoptosis [50]. The MAPK pathway could transmit signals to the nucleus to activate a large number of transcription factors, many of which could regulate SC myelination by activating a large number of genes and enzymes related to myelination in SCs. In addition, the activated Ras pathway could directly interact with PI3K, while PI3K could activate the downstream Erk pathway. These signaling pathways interacted with each other to form a complex microenvironment that regulated cell development.

## Conclusions

In this study, a DA-modified CS/AS scaffold with surface anisotropic structure was prepared by micro-molding technology. The topological structure could guide the orientation growth of DRG axons, SCs and ECs, and the combination of topological structure and ECs/SCs co-culture systems effectively guided the growth of neuronal axons and prevented entanglement of axons, indicating a certain inhibitory effect on neurofibromas. In addition, the combination of topological structure and cell co-culture system could significantly up-regulate the expression of genes related to myelination, cell proliferation and cytoskeleton rearrangement via PI3K-Akt, MAPK and cytokine and receptor chemokine pathways, and simultaneously promote the normal secretion of various neurotropic factors. In summary, the co-culture of SCs and ECs with neuronal cells on DA-modified topological biomaterial scaffolds showed potential application prospects for peripheral nerve regeneration. This study may provide an important experimental basis for the design and development of nerve implants.

## Abbreviations

CS/AS: Chitosan/artemisia sphaerocephala; DA: Dopamine; DAPI: 4′,6-Diamidino-2-phenylindole; DMEM: Dulbecco’s modified Eagle’s medium; DRG: Dorsal root ganglion; EC: Epithelial cell; Erk: Extracellular signal-regulated kinase; FBS: Fetal bovine serum; IkB: Inhibitor of nuclear factor kappa-B; MAPK: Mitogen-activated protein kinase; NGF: Nerve growth factor; OM: Optical microscopy; PBS: Phosphate-buffered saline; PI3K: Phosphoinositide 3-kinase–protein kinase B; SC: Schwann cell; SEM: scanning electron microscopy; VEGF: Vascular endothelial growth factor.

## Author contribution

TZ: Methodology, Validation, Software, Investigation, TZ, GL: Investigation, Formal analysis, Writing - review & editing, LW, SS, QH, JX, YL: Formal analysis, Visualization, RW, GL: Conceptualization, Writing - original draft, Writing - review & editing, Supervision, Project administration.

## Conflicts of interest

On behalf of all authors, the corresponding author states that there is no conflict of interest.

## Ethics approval

All experimental operations were carried out and data were collected following the standards of the Animal Ethics Committee of the Nantong University.

## Funding

The study was supported by the National Natural Science Foundation of China (32171352, 82001295), Natural Key Science Research Program of Jiangsu Education Department (19KJA320006), Open Project of Guangxi Key Laboratory of Regenerative Medicine (Guizaizhongkai 202101) and Opening Project of National Engineering Laboratory for Modern Silk, Soochow University (SDGC2147) and Jiangsu Provincial Double-Innovation Doctor Program (JSSCBS20211120).

## Supplementary Material

Revised_Manuscript-Supporting_Information_tkac030
