# Supplementary material for: Co-culture of Schwann cells and endothelial cells for synergistically regulating dorsal root ganglion behavior on chitosan-based anisotropic topology for peripheral nerve regeneration
Source: Burns Trauma. 2022 Sep 4;10:tkac030. doi: 10.1093/burnst/tkac030 (PMC9444262; doi:10.1093/burnst/tkac030)
Supplement: Revised_Manuscript-Supporting_Information_tkac030 [file revised_manuscript-supporting_information_tkac030.docx]

Supporting Information

**Co-culture of Schwann cells and Endothelial Cells for Synergistically Regulating** **Dorsal Root Ganglion Behavior on Chitosan-Based Anisotropic Topology for Peripheral Nerve Regeneration**

**Manuscript Type: Article**

**The number of pages, figures, and tables: five pages****, two figures and one table.**

**1.Supplementary Figures and Table**


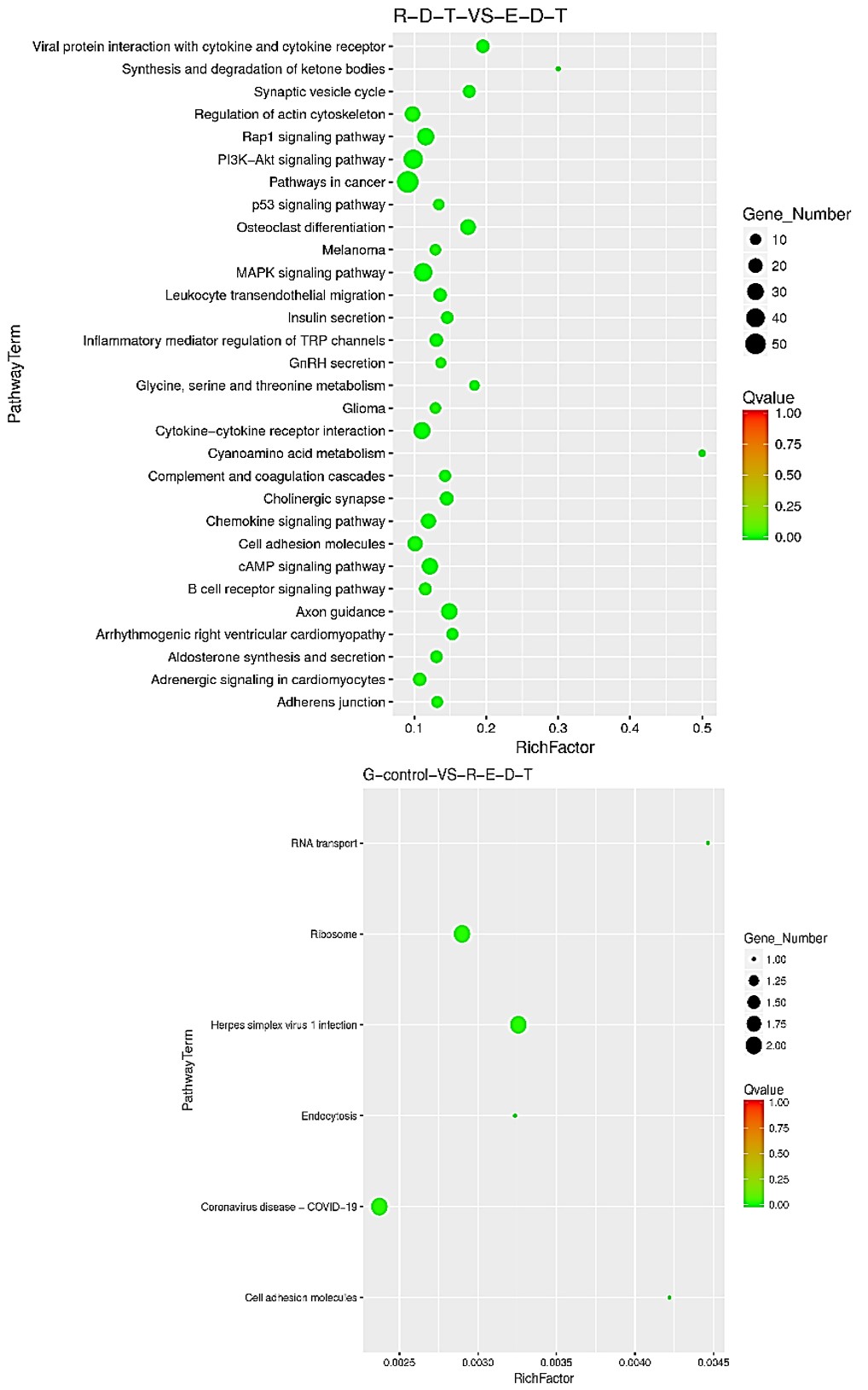

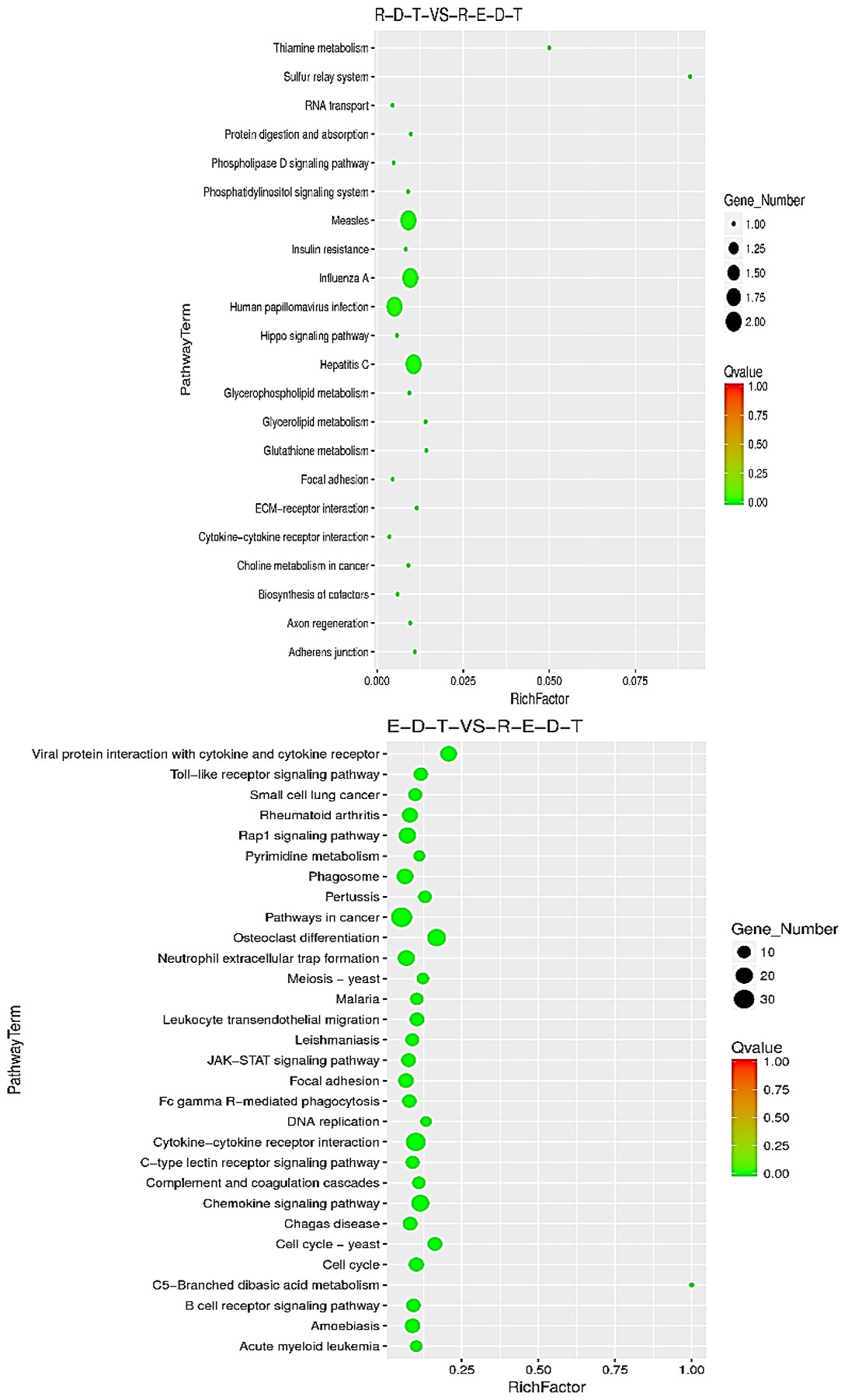


**Figure S1. Differential gene KEGG enrichment scatter plot. The size of the dot indicates the number of differentially expressed genes in this pathway, and the color of the dot corresponds to different Qvalue ranges.** ***R* RSC96 rat Schwann cells, *E* endothelial cell, *D* dorsal Root Ganglion, *T* topological structure**

**
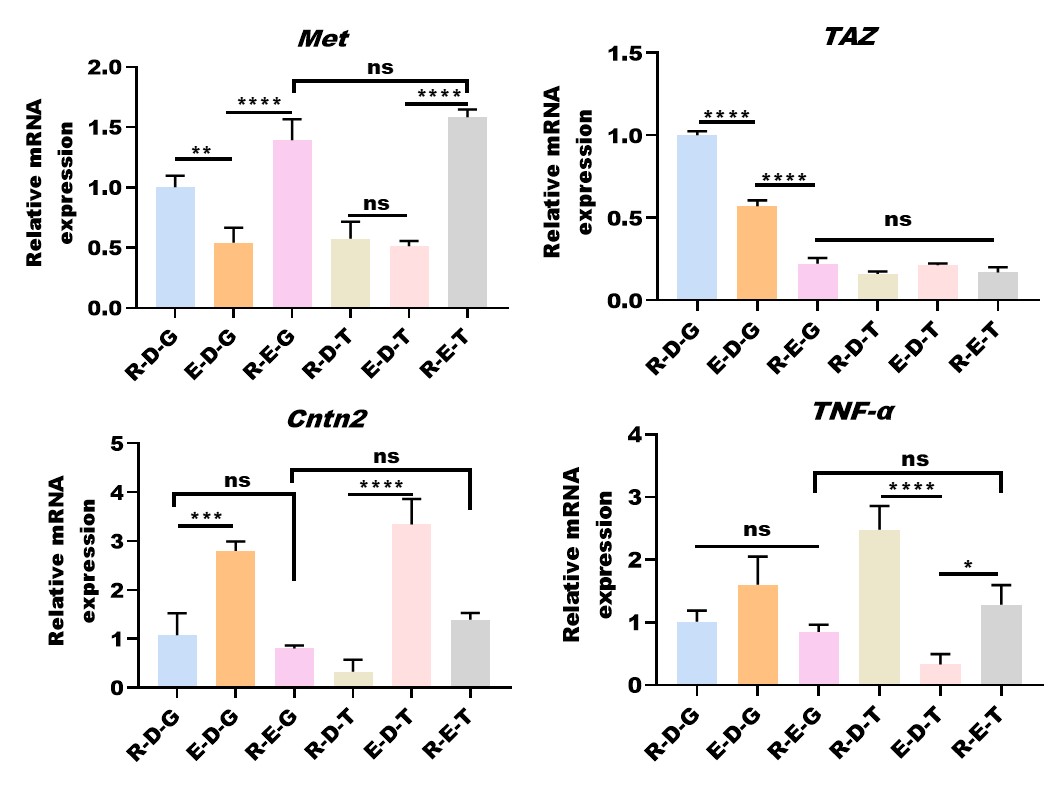
**

**Figure S2. Gene expression detected by qRT-PCR.** **The method of one-way analysis of variance is used. Data are shown as means ± SD. Statistical analysis: *p<0.05, **p<0.01, *** p <0.001, **** p <0.0001, ns no significant difference. *R* RSC96 rat Schwann cells*, E* endothelial cell*, D* dorsal Root Ganglion*,* *G* glass*,* *T* topological structure*, Met* Mesenchymal-epithelial transition factor*, TAZ* tafazzin*, Cntn2*** **contactin2*, TNF-α* tumor necrosis factor-α**

**Table S1. Primer sequences for qPCR.**

| **Gene names** | **Forward primer (5′-3′)** | | **Reverse primer (5′-3′)** |
| --- | --- | --- | --- |
| Cntn2  MPZ  Met  TNF-α  EGR2  Sox10  YAP  TAZ  GAPDH | | GGGGTGTGGTGGTGAGA  GACAACGGCACTTTCACAT  GTTCGCTATGACGCAAGAG  TGCTCAGAAACACACGAGA  CAGATGAACGGAGTGGCT  GGAGAGGTCGGAGGAGGT  CAGGCCAGTACCGATGC  TCGTCATGGGTCTAGTTGG  TCTCTGCTCCTCCCTGTTC | CCGAGGGTGGAGTACGAG  CAACACCACCCCATACCT  AGAGGAGTTGGGAAACTGG  ATCCACTCAGGCATCGAC  CTAGGCGCAGAGATGGG  GGACAGGCAGCGAGGTT  CGGGCCAGAGACAACAC  GTGATAAGAGGGGTCGCA  ACACCGACCTTCACCATCT |

Cntn2 contactin2, MPZ myelin protein zero, Met Mesenchymal - epithelial transition factor, TNF-α tumor necrosis factor-α, EGR2 early growth response 2, Sox10 SRY-box transcription factor 10, TAZ tafazzin, YAP yes-associated protein
